# Delivery of A Jagged1-PEG-MAL hydrogel with Pediatric Human Bone Cells Regenerates Critically-Sized Craniofacial Bone Defects

**DOI:** 10.1101/2023.10.06.561291

**Published:** 2023-10-10

**Authors:** Archana Kamalakar, Brendan Tobin, Sundus Kaimari, Afra I. Toma, Irica Moriarity, Surabhi Gautam, Pallavi Bhattaram, Shelly Abramowicz, Hicham Drissi, Andrés J. García, Levi B. Wood, Steven L. Goudy

**Affiliations:** 1 Department of Pediatric Otolaryngology, Emory University, Atlanta, GA, USA; 2 Department of Cell biology, Emory University, Atlanta, GA, USA; 3 Department of Orthopaedics, Emory University, Atlanta, GA, USA; 4 Department of Surgery, Division of Oral and Maxillofacial Surgery, Emory University, Atlanta, GA, USA; 5 Department of Pediatric Otolaryngology, Children’s Healthcare of Atlanta, Atlanta, GA, USA; 6 Wallace H. Coulter Department of Biomedical Engineering, Georgia Institute of Technology, Atlanta, GA, USA; 7 Neuroscience Program in College of Sciences, Georgia Institute of Technology, Atlanta, GA, USA; 8 Parker H. Petit Institute for Bioengineering and Biosciences, Georgia Institute of Technology, Atlanta, GA, USA; 9 George W. Woodruff School of Mechanical Engineering, Georgia Tech College of Engineering, Atlanta, GA, USA; 10 The Atlanta Veterans Affairs Medical Center Atlanta, GA, USA; 11 School of Chemistry and Biomolecular Engineering, Georgia Tech College of Engineering, Atlanta, GA, USA

**Keywords:** JAGGED1, Human Bone-derived Osteoblast-like Cells, PEG-4MAL Hydrogel-based delivery, Craniofacial Bone Defects, Bone Regeneration, Non-canonical JAG1-NOTCH pathways, RNA Sequencing

## Abstract

Treatments for congenital and acquired craniofacial (CF) bone abnormalities are limited and expensive. Current reconstructive methods include surgical correction of injuries, short-term bone stabilization, and long-term use of bone grafting solutions, including implantation of (i) allografts which are prone to implant failure or infection, (ii) autografts which are limited in supply. Current bone regenerative approaches have consistently relied on BMP-2 application with or without addition of stem cells. BMP2 treatment can lead to severe bony overgrowth or uncontrolled inflammation, which can accelerate further bone loss. Bone marrow-derived mesenchymal stem cell-based treatments, which do not have the side effects of BMP2, are not currently FDA approved, and are time and resource intensive. There is a critical need for novel bone regenerative therapies to treat CF bone loss that have minimal side effects, are easily available, and are affordable. In this study we investigated novel bone regenerative therapies downstream of JAGGED1 (JAG1).

We previously demonstrated that JAG1 induces murine cranial neural crest (CNC) cells towards osteoblast commitment via a NOTCH non-canonical pathway involving JAK2-STAT5 (1) and that JAG1 delivery with CNC cells elicits bone regeneration *in vivo*. In this study, we hypothesized that delivery of JAG1 and induction of its downstream NOTCH non-canonical signaling in **pediatric human osteoblasts** constitute an effective bone regenerative treatment in an *in vivo* murine bone loss model of a critically-sized cranial defect. Using this CF defect model *in vivo*, we delivered JAG1 with pediatric human bone-derived osteoblast-like (HBO) cells to demonstrate the osteo-inductive properties of JAG1 in human cells and *in vitro* we utilized the HBO cells to identify the downstream non-canonical JAG1 signaling intermediates as effective bone regenerative treatments. *In vitro*, we identified an important mechanism by which JAG1 induces pediatric osteoblast commitment and bone formation involving the phosphorylation of p70 S6K. This discovery enables potential new treatment avenues involving the delivery of tethered JAG1 and the downstream activators of p70 S6K as powerful bone regenerative therapies in pediatric CF bone loss.

## 1. Introduction:

Craniofacial (CF) injuries comprise more than 25% of injuries reported to the National Trauma Data Bank in the US every year (2–4). Left untreated, CF injuries can severely impair critical daily functions related to breathing, speech, eating, and swallowing, thus requiring urgent repair (5). Current methods to repair CF bone loss include the direct implantation of either allografts or autografts, and/or subsequent revision surgeries (6, 7). Although generally successful, these conventional treatments present several limitations. Some bone donor sites are rib, fibula, iliac crest, scapula, distal tibia, and medial femoral condyle. All of these donor sites have limited availability and are not anatomically similar to the bone they replace; osteotomies are required to shape them so that they resemble the shape of the CF bones (8, 9). The allograft survival rate following an iliac graft procedure is 96.1% and the risk of infection ranges from 5 to 33% (7, 10). For maxillary or mandibular bone replacement, the iliac crest is the preferred graft source due to its robust corticocancellous anatomy. Risks associated with iliac crest grafting include significant pain at the donor site, nerve injury, decreased load bearing on the ipsilateral leg, and increased risk of hip fractures (11, 12). Due to the limited supply of bone, revision bone graft surgeries are often required and are expensive ($35,000 – $52,000 per patient) and cause great discomfort (13). These and other complications undermine the patient’s quality of life due to the social stigma resulting from the facial deformity, which can also lead to psychological distress (14).

Craniofacial bone development primarily occurs through intramembranous ossification, a process where pre-osteoblasts mineralize directly, without a cartilage intermediate, making it distinct from long bone development (endochondral ossification) (15). Intramembranous ossification recruits Cranial Neural Crest (CNC) cells as osteoblast precursors during CF bone development (15). Extensive efforts have been made towards using Parathyroid Hormone (PTH (1–34)), Vascular Endothelial Growth Factor (VEGF), Fibroblast Growth Factor (FGF), Stromal Cell-Derived Factor-1 (SDF-1) or Transforming Growth Factor- Beta 2 (TGFβ2) as alternatives to bone regenerative treatments in preclinical models but resulted in limited success as an *in vivo* treatment option (16–20). Platelet-rich Plasma (PRP) supplemented with various biological growth factors such as VEGF, FGF, and TGFβ2 have also been used to enhance wound healing, chemotaxis, angiogenesis, proliferation of mesenchymal stem cells and osteoblasts. Many of these studies demonstrated potential improvement in bone healing; however, these strategies face significant translational barriers (20). Bone Morphogenetic Protein-2 (BMP2) is an FDA-approved bone regenerative strategy along with the delivery of stem cells. BMP2 is used to reconstruct spine and grafted maxillary bone in adults (21, 22). BMP2 treatment can lead to ectopic bone growth, hypertrophy and life-threatening side effects (e.g., uncontrolled inflammation), which may accelerate bone loss (23, 24). The use of BMP2 for the treatment of pediatric cases of CF trauma is not FDA-approved due to concerns of severe swelling of the face and airways. Although generally considered safer, stem cell-based treatments are time consuming, have heterogenous results and add to the high expense of repairing CF bone loss (22). *Thus, there is a critical need for novel bone regenerative therapies to treat CF bone loss that have minimal side effects, are readily accessible and affordable*.

The NOTCH signaling pathway is involved in many cellular processes, including determination of cell fate, and has been explored as a potential target for regeneration of long bone injuries (25). NOTCH signaling occurs via cell-to-cell binding of a NOTCH ligand (e.g., JAG1) to a NOTCH receptor. Internalization of the NOTCH intracellular domain leads to the expression of canonical NOTCH genes *Hes1* and *Hey1*, which are known to have both osteo-inductive and osteo-inhibitory roles, thus obfuscating the effectiveness of NOTCH-based bone regenerative therapies (26, 27). However, we and others have demonstrated that JAG1 exhibits osteo-inductive properties, as demonstrated by induction of pre-osteoblast genes like *Runx2* in CNC cells, even when the canonical NOTCH pathway is inhibited (1, 28). Our recent publications further identified a JAG1-JAK2 non-canonical signaling pathway that promotes murine CNC commitment to osteoblast differentiation in mice (1).

This unexpected finding raises critical questions about the role of non-canonical JAG1 signaling during human craniofacial regeneration. ***In this study we postulated that 1) JAG1 can induce osteoblast differentiation and mineralization of pediatric Human Bone-derived Osteoblast-like (HBO) cells, and 2) the delivery of JAG1 non-canonical signaling constitutes an effective treatment for inducing bone regeneration in a pediatric, preclinical craniofacial bone loss model*.** Consequently, we evaluated (i) the ability of JAG1 to regenerate bone in a pediatric critically-sized CF defect murine model when delivered in a synthetic hydrogel coupled with pediatric HBO cells, and (ii) characterized the downstream JAGGED1 non-canonical signaling mechanisms. Our results reveal potential treatment options in the form of JAG1 and/or its downstream targets to induce bone regeneration in CF bone loss injuries and provide alternative treatment options for CF defects.

## 2. Materials & Methods:

### 2.1 HBO cell isolation:

HBO cell lines were derived from healthy fibulas of seven pediatric subjects under appropriate Institutional Review Board approval, Segments of the fibulas were digested using Collagenase A (0.1 mg/mL) treatment for 40 minutes with replacement of Collagenase A midway at 20 minutes and then digested with 0.2 mg/ml of Collagenase A for 60 minutes at 37°C with intermittent shaking. The segments of bone were maintained in DMEM + Primocin + 10% FBS + 50 μM ascorbic acid (Sigma, 49752) + 10nM dexamethasone. Media changes were done every 5 days. Osteoblast-like cells began to grow out of the bone segments by Day 10. These cells can be passaged and frozen for storage. On addition of Osteogenic media (DMEM + Primocin + 10% FBS + 50 μM ascorbic acid + 10 nM dexamethasone + 10 mM beta-glycerophosphate disodium (Sigma, G9422), the cells form mineralized nodules indicating their osteogenic ability as seen in **Supplementary Figure 1**.

### 2.2 JAG1 immobilization:

As per manufacturer’s recommendations, 50 μL (1.5 mg) of Dynabeads Protein G (Invitrogen 10004D) were transferred to a tube, where the beads were separated from the solution using a magnetic tube rack (Biorad, 1614916) and washed once using 200 μL PBS with 0.1% Tween-20 (Fisher, BP337-500). The wash buffer was separated from the beads-Fc complex using the magnetic rack. Recombinant JAG1-Fc (5 μM, 5.7 μM, 10 μM or 20 μM) (Creative Biomart, JAG1-3138H) or control IgG-Fc fragment (5 μM or 5.7 μM) (Abcam, ab90285) were diluted in 200 μL PBS with 0.1% Tween-20 and then added to the Dynabeads. The beads plus proteins were incubated at 4°C with rotation for 16 hours. Thereafter, the tubes were placed back on the magnetic rack and the supernatant was removed. The bead-JAG1/Fc complex was resuspended in 200 μL PBS with 0.1% Tween-20 to wash by gentle pipetting. The wash buffer was also separated from the beads-Fc complex using the magnetic rack, and the final suspension of the beads in hydrogels was used as treatment.

### 2.3 Alizarin Red assay:

HBO cells were seeded at 30,000 cells per well in a 12-well plate, treated (n = 3) with and cultured for 21 days in osteogenic media (DMEM + Primocin + 10% FBS + 50 μM ascorbic acid + 10 nM dexamethasone + 10 mM beta-glycerophosphate) with half feeds every 5 days. Treatments included growth media (DMEM + Primocin + 10% FBS), osteogenic media, Fc-Dynabeads (5.7 μM) in osteogenic media or JAG1-Dynabeads (5.7 μM) in osteogenic media. On day 21, the cells were fixed using 50% ethanol for 15 minutes at 4°C. The fixed cells were then stained with Alizarin Red S dye to detect mineralization. The dye was extracted using a 10% acetic acid solution in water and quantified by measuring the absorbance at 420nm using a spectrophotometer.

### 2.4 Hydrogel preparation:

We prepared poly(ethylene glycol) (PEG)-based synthetic hydrogels incorporating cell adhesive peptides in two steps. First, maleimide end-functionalized 20 kDa four-arm PEG macromer (PEG-4MAL, with > 95% end-group substitution, Laysan Bio, 4ARM-PEG-MAL-20K), was reacted with a thiol-containing adhesive peptide GRGDSPC (Genscript, RP20283) in PBS with 20 mM HEPES at pH 7.4 for 1 hour. Then, the RGD-functionalized PEG-4MAL macromers were cross-linked in the presence of HBO cells and JAG1-Dynabeads into a hydrogel by addition of the dithiol protease-cleavable peptide cross-linker GPQ-W (GCRDGPQGIWGQDRCG) (New England Peptides, Inc, (NEP) Custom synthesized) (1, 29). The final gel formulation consisted of 4.0% wt/vol polymer and 1.0 mM RGD.

### 2.5 *In vivo* experiments:

We performed all *in vivo* experiments using procedural guidelines with appropriate approvals from the Institutional Animal Care and Use Committee of Emory University. 6-8-week-old male and female NOD-SCID mice (The Jackson Laboratory, 001303) were used. As shown in **supplemental figure 3**, the surgery site was disinfected and then incisions were made using sterile surgical equipment to expose the parietal bones of the mice. Thereafter, 4 mm defects were created in the parietal bones using a variable speed drill (Aseptico (MicroNX), MAX-88ESP, CL1791023) and sterile circular knives. **JAG1 Delivery:** PEG-4MAL hydrogels (20 μL) loaded with JAG1-Dynabeads without or with 100,000 HBO cells (n = 13 - 15 for all treatment groups) were placed within the defects created in parietal bones in the NOD-SCID mouse skulls as the first dose. A second dose of the hydrogels encapsulating HBO cells and Dynabead-bound JAG1 were administered as transcutaneous injections during week 4 to continue the bone regenerative action of JAG1 and the skulls were then harvested at week 8, fixed using 10% neutral buffered formalin (VWR, 89370-094) and subjected to micro computed tomography (μCT).

### 2.6 Micro Computed Tomography (μCT):

μCT analyses were conducted according to current guidelines for the assessment of bone volume within the defects created in mouse calvaria (30). Briefly, formalin-fixed skulls were positioned in the μCT tubes with the nose facing the bottom of the tube and imaged in a μCT 40 (Scanco Medical AG, Bassersdorf, Switzerland) using a 36 μm isotropic voxel size in all dimensions. Thereafter, using a consistent and pre-determined threshold of 55 kVp, 145 μA, 8 W and 200 ms integration time for all measurements, three-dimensional (3D) reconstructions were created by stacking the regions of interest from ~600 two-dimensional (2D) slices consisting of the entire skull and then applying a gray-scale threshold of 150 and Gaussian noise filter (σ=0.8, support=1.0), a coronal reformatting was done. Thereafter, a circular region of interest (ROI) encompassing the defect was selected for analysis consisting of transverse CT slices encompassing the entire defect and, new bone volume (BV) was calculated.

### 2.7 Histology:

Formalin-fixed skulls used for MicroCT measurements were decalcified in Cal-ex (Fisher, C5510-1D), embedded in paraffin, and sectioned on a microtome to obtain 5 μm thick sections which were then stained using a Masson Trichrome staining kit (Sigma Aldrich, HT15) and according to the manufacturer’s protocol. The sections were first washed with PBS, three times, for 5 minutes each, then the slides were submerged in Bouin’s solution for 15 minutes, and then washed under running water for 5 minutes. Thereafter, the sections were incubated in Weigert’s working hematoxylin solution for 10 minutes before being washed three times under running water for 5 minutes each wash and then transferred to distilled water. The sections were then stained with biebrich scarlet acid fuchsin for 5 minutes and washed with distilled water three times before being submerged in a phosphotungstic/phosphomolybdic solution for 10 minutes, and subsequently placed in an analine blue solution. Lastly, they were washed with distilled water three times and moved to a solution of 1% acetic acid for 1 minute followed by a submersion in two changes of xylene and mounted with a coverslip. The sections were then imaged using brightfield microscopy.

### 2.8 RNA Sequencing:

RNA was isolated using the Qiagen RNeasy kit (Qiagen, 74106) according to manufacturer’s protocols. The samples were submitted to the Molecular Evolution core at the Georgia Institute of Technology for sequencing. Quality Control (QC) was performed using an Agilent Bioanalyzer 2100 to determine the RNA Integrity Number (RIN) of the samples. mRNA was enriched using the New England Biolab’s (NEB) NEBNext Poly(A) mRNA isolation module for samples with RINs greater than 7 and libraries were prepared using the NEBNext Ultra II directional RNA library preparation kit (NEB, E7760). QC was then performed on these libraries using Agilent Bioanalyzer 2100 and the libraries were quantified using fluorometric methods. Paired-end 150 base pairs (PE150) sequencing was performed on the Illumina NovaSeq 6000 instrument to obtain a sequencing depth of 30 million reads per sample. The transcripts obtained were aligned using the hg38 genome reference database along with elimination of duplicate reads, using the DNAStar Lasergene 17.3 application. The RNA levels were calculated in reads per kilobase per million mapped reads (RPKM). Genes expressed at > 1.5 RPKM were retained for further analyses.

### 2.9 Transcriptomic Analysis Methods:

#### 2.9.1 Differential Gene Expression and Enrichment Analysis:

Differentially expressed genes (DEGs) were determined using DESeq2 (v1.38.3) available in R Bioconductor (31). Transcripts with an FDR-adjusted p-value<0.05 were considered significant for this analysis. Transcript counts were normalized using the median-of-ratios method used by DESeq2 prior to differential expression analysis. Results were visualized with venn diagrams using govenn (v0.1.10), volcano plots in R using ggplot2 (v3.4.1) (H. Wickham. ggplot2: Elegant Graphics for Data Analysis. Springer-Verlag New York, 2016.) and heatmaps in R using Heatmap3 (v1.1.9) (32).

#### 2.9.2 Transcript Functional Annotation:

To provide additional functional annotation of the DEGs, a web-scraper was built to parse the National Center for Biotechnology Information Gene Database (33) entry for each transcript and identify relevant terms. Transcript names were connected to the NCBI Entrez ID with the Genome wide annotation for Human package, org.Hs.eg.db (v3.15.0), available through Bioconductor in R Carlson M (2019). *org.Hs.eg.db: Genome wide annotation for Human*. R package version 3.15.0. This tool utilized the HTML parser in the BeautifulSoup4 (v4.12.2) Python package to extract the gene summary information from each gene entry in the database. The script then identified the keywords in the summary.

#### 2.9.3 Functional Over-Representation Analysis:

Over-represented Gene Ontology (GO) terms were identified using PANTHER (v17.0) (34, 35) DEGs involved in the noncanonical signaling pathway were identified from Venn diagrams and separated by up-regulated and down-regulated relative to control. Each list was uploaded to the PANTHER online tool for over-representation testing. FDR-adjusted Fischer’s test p<0.05 was considered over-represented in this analysis. The complete GO Biological Process term list was evaluated and the complete list of identified transcripts from RNAseq was set as the background.

### 2.10 Luminex-based Multiplex assay:

To detect phospho-signaling targets, serum-starved HBO cell lines (N = 3) from different patients were treated (n = 3 per cell line) with Fc-Dynabeads (5.7 μM), unbound BMP2 (100 nM), and JAG1-dynabeads (5.7 μM) with or without N-[N-(3,5-Difluorophenacetyl)-L-alanyl]-S-phenylglycine t-butyl ester (DAPT) (15 μM) as a time course stimulation for 5, 10, 15, and 30 minutes. Whole cell protein (2 μg) lysates were subjected to a Millipore Luminex based Multiplex assay to measure signaling targets using the Milliplex multiple pathway cell signaling magnetic bead 9-Plex kit (Millipore Sigma, 48-681 MAG) according to manufacturer’s protocol.

### 2.11 Statistics:

Data were analyzed by analysis of variance (ANOVA) with Tukey’s post-hoc test using GraphPad Prism 8. All data are presented as mean ± SD. p < 0.05 between groups was considered significant and are reported as such. MATLAB coding was used to create heatmaps and to generate z-score values associated with color intensities seen on the heatmap.

## 3. Results:

### JAG1 induces mineralization of pediatric human bone derived osteoblast-like cells:

**Figure 1:**
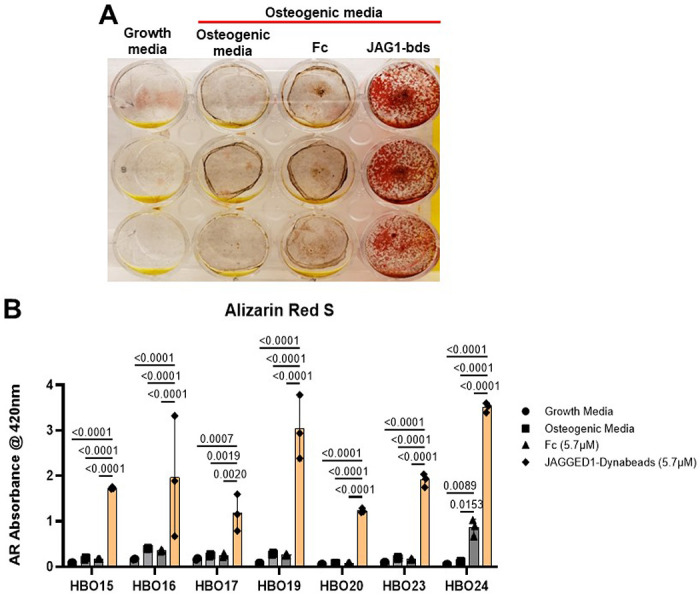
JAGGED1-induced mineralization of HBO cells: Seven HBO cell lines were treated with growth media alone, osteogenic media alone or with Fc (5.7 μM) or JAG1 (5.7 μM). The cells were half-fed every 5 days. On day 21 cells are fixed with 50% ethanol and thereafter, stained with 1% Alizarin Red S. (B) Alizarin Red S dye was extracted from Alizarin Red S-stained cells using a 1:10 dilution of acetic acid and water, and the absorbance was read at 420 nm. Data represents the mean values of three technical replicates per cell line (mean ± SD, one way ANOVA with Tukey post-hoc).

To test whether JAG1 induces osteoblast commitment and differentiation in human osteoblast-like primary cells, we derived human bone osteoblast-like (HBO) cell lines (HBO15, 16, 17, 19, 20, 23, 24) from seven healthy pediatric human bone samples, as described (36) (**Suppl Fig. 1**). HBO cells were then treated with Fc-Dynabeads (5.7 μM) as a negative control, since JAG1 is a chimeric recombinant protein with an Fc-portion, and JAG1-Dynabeads (5.7 μM), in the presence of osteogenic media. On day 21, the cells were stained for mineralization using Alizarin Red S stain (Fig. 1A & 1B). We observed significantly increased mineralization in JAG1-treated samples compared to growth media-treated, osteogenic media-treated, and Fc-Dynabead-treated (p = 0.0107) cells. These data indicate that JAG1 can induce osteoblast commitment, differentiation, and mineralization of pediatric HBO cells.

### Delivery of JAG1-Dynabead-PEG-4MAL with pediatric HBO cells repairs critically-sized cranial defects:

**Figure 2:**
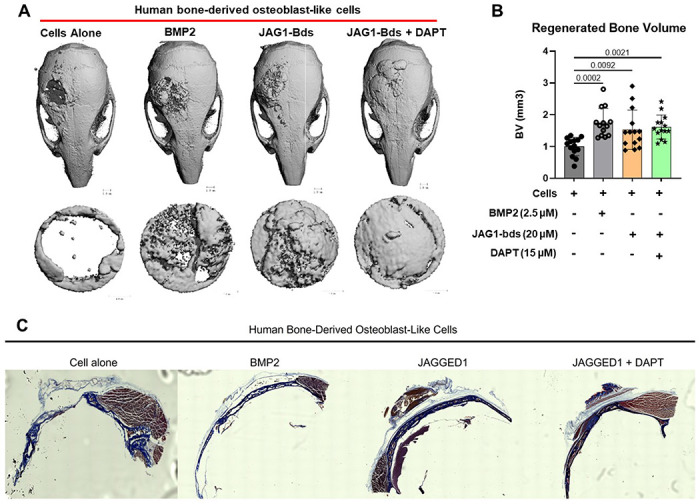
JAG1 delivery in a PEG hydrogel stimulates bone regeneration in a critical-sized bone defect mouse model: HBO cells alone or in the presence of JAG1-Dynabeads complex (20 μM) ± DAPT and BMP2 (2.5 μM)+ Fc-Dynabeads were incorporated in 4% PEG-MAL hydrogels and implanted into 4 mm critical-sized defects in the parietal bones of 6-8-week old NOD SCID mice (n = 4 – 6 per HBO-cell donor, 13 – 15 total) as 2 separate doses (Initial dose, Week 4). After 8 weeks, we quantified differences in regenerated bone volume, as seen in B, within the defect and compared them between experimental groups by μCT analysis. μCT reconstructions of defects are shown in A. Data are presented as mean (n = 13 - 15) ± SD with p-values reported. C shows representative sections of the defect area on skulls from mice from all experimental groups stained with Masson Trichrome stain.

Since we observed an induction of osteoblast commitment and differentiation in pediatric HBO cells *in vitro*, we next assessed whether co-delivery of JAG1-presenting hydrogels with pediatric HBO cells act similarly in murine craniofacial defects. We recently reported that JAG1 can induce murine CNC-cell osteoblast commitment and repair cranial bone defects *in vivo* (37). To establish a more translatable use of JAG1 in treating CF bone loss, we tested whether JAG1 can stimulate human cells (pediatric HBO) to facilitate bone regeneration in NOD-SCID mice, to prevent graft rejection. JAG1-Dynabead-PEG-4MAL hydrogels also encapsulating pediatric HBO cell obtained from 3 separate donors were implanted in critically-sized parietal bone defects (4 mm) in NOD-SCID mice (n = 4 – 6 per donor, 13 – 15 total) (**Suppl Fig. 3**). The volume of bone regenerated by the JAG1-PEG-4MAL-pediatric HBO hydrogel with or without DAPT, an inhibitor of NOTCH canonical signaling, was measured, and compared to HBO cells alone, Fc-Dynabead (20 μM) + BMP2 (2.5 μM) treatments. To maintain continuous osteogenic induction, additional JAG1-PEG-4MAL-pediatric HBO cells and control hydrogels were injected into the defects transcutaneously at week 4. After 8 weeks, we quantified differences in bone volume (BV) within the cranial defect using μCT analysis (Fig. 2). We observed that there was minimal bone regenerated in mice treated with cells alone. As expected, BMP2 significantly increased regenerated bone volume (p = 0.0002) compared to the cells alone group. The bone volume regenerated by JAG1 in the absence (fold change: 1.5) and presence of DAPT (fold change:1.6) was significantly higher compared to the cells alone treatment group (p = 0.0092, 0.0021 respectively). In Figure 2C, Paraffin sections of mouse skulls were stained with Masson trichrome stain, as described in methods under histology. Qualitative assessments of the stained sections revealed increased collagen (blue color) in samples obtained from mice treated with BMP2, as expected, JAG1 and JAG1 + DAPT compared to the cells alone treated mice. This suggests that JAG1 can be used as a bone regenerative therapy where JAG1 induces bone regeneration independently of NOTCH canonical signaling in human cells.

### Transcriptional profiling of cultured HBO cells reveals genes regulated by the non-canonical NOTCH pathway:

**Figure 3:**
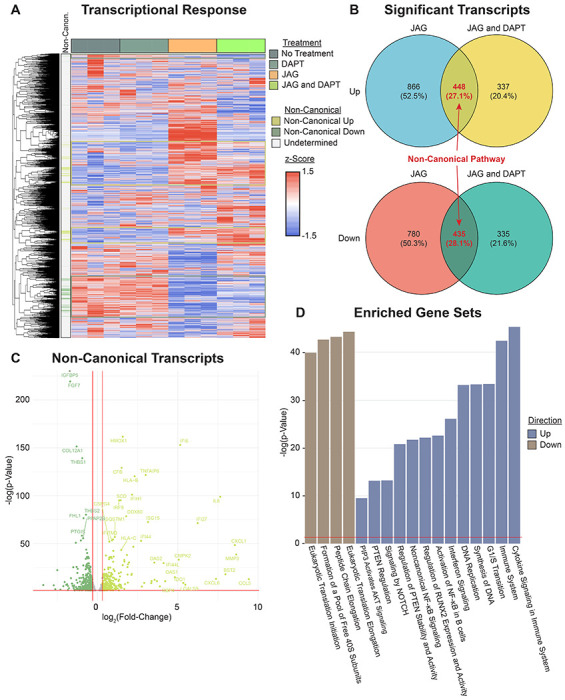
Transcriptional profiling reveals genes and pathways stimulated by non-canonical NOTCH signaling. (**A**) RNAseq reveals clusters of genes associated with the non-canonical NOTCH pathway (rows are z-scored, side color bar identifies up- and down-regulated DEGs stimulated by the non-canonical pathway). (**B**) Overlapping DEGs in the JAG1 vs no-treatment and JAG1+DAPT vs no-treatment comparisons reveal the non-canonical pathway (DEseq2). (**C**) Overlapping DEGs from JAG1+DAPT vs no-treatment comparison. (**D**) Gene ontology over-representation test reveals significantly enriched up- and down-regulated pathways (FDR adjusted).

We previously found that JAG1 can activate a NOTCH non-canonical JAK2-STAT5 signaling pathway in mouse CNC cells, which stimulated expression of osteoblast genes (Runx2 and Osteocalcin) as well as osteoblast commitment and proliferation (1, 37). Thus, we asked if JAG1 would have similar effects for a non-canonical NOTCH pathway on HBO cells. To test this, we cultured HBO cells from a single donor in triplicate and conditioned for 24 hours with vehicle, DAPT, JAG1, or both. Comparison of the JAG1 and JAG1+DAPT conditions revealed clusters of genes that were up- or down-regulated by JAG1 stimulation and remained with inhibition of NOTCH, i.e., defining the non-canonical pathway (Figure 3A). Analysis of differentially expressed genes (DEGs) in JAG1 and JAG1+DAPT groups compared to no treatment revealed a total of 448 up-regulated genes and 435 down-regulated genes in the non-canonical pathway (Figure 3B, **Supplementary Table 1**). These include up-regulation of genes involved in osteoblast commitment (RUNX2), matrix remodeling (MMP3), and diverse cytokines and chemokines (CCL5, CXCL1, CXCL6) as part of the non-canonical pathway (Figure 3C). Gene ontology analysis of the up-regulated genes in the non-canonical pathway revealed significant over-representation of GO Terms associated with RUNX2, cytokine signaling, NF-κB, and cell cycle (Figure 3D). More interestingly, the PIP3 activating AKT signaling pathway was upregulated by JAG1 treatment, suggesting that JAG1 can activate NOTCH non-canonical signals via the AKT pathway.

Collectively, these data suggest that JAG1 has a profound NOTCH non-canonical effect on HBO cells that stimulates HBO cell-osteoblast commitment and differentiation leading to HBO-cell induced bone formation.

### JAG1 induces increased cytokine production and phosphorylation of NOTCH non-canonical pathway targets in pediatric HBO cells:

**Figure 4:**
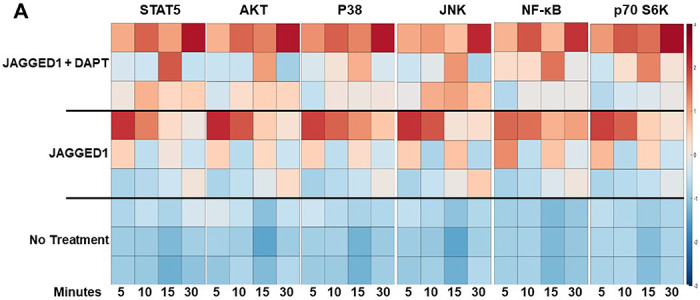

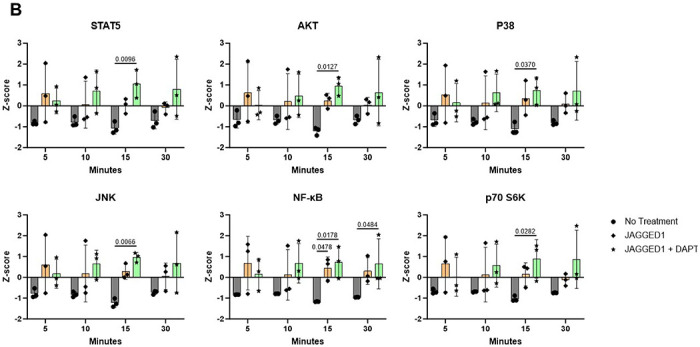
JAGGED1 induces a non-canonical NOTCH pathway in HBO cells. HBO cells undergo mineralization through non-canonical pathway. Luminex analysis of lysates obtained from three HBO cell lines untreated or treated with Dynabeads-bound recombinant JAG1-Fc fragment (5.7 μM) ± DAPT (15 μM), a NOTCH canonical pathway inhibitor in a time course manner (5, 10, 15 and 30 minutes), revealed that STAT5, AKT, P38, JNK, NF-κB and p70 S6K phosphorylation was increased by JAG1 + DAPT treatment as shown by A) heatmaps and B) Z-scores plotted on graphs. Each data point represents mean n = 3 ± SD per cell line with p-values reported.

Having observed that JAG1 induced osteoblast commitment of murine CNC cells via a NOTCH non-canonical pathway (JAG1-JAK2), we sought to determine whether JAG1 activated NOTCH non-canonical pathways and targets in the pediatric HBO cells. Serum-starved pediatric HBO cells were subsequently treated with JAG1-Dynabeads (5.7 μM) with or without DAPT (15 μM), to block the NOTCH canonical pathway, as a time course stimulation for 5, 10, 15, and 30 minutes. Phosphorylation levels for signaling molecules were assessed via Luminex-based multiplex assays. We observed significantly increased phosphorylation of multiple signaling molecules, including STAT5, AKT, P38, JNK, NF-κB, and p70 S6K in JAG1-treated cells, even in the presence of DAPT (Fig. 4). We have previously shown that JAG1 induced the phosphorylation of STAT5 during CNC cell differentiation to osteoblasts (37). Prior studies emphasize the importance of non-canonical signaling, crosstalk between NOTCH, and other cellular signaling mechanisms. For example, the WNT pathway cross-talks with the NOTCH canonical pathway during vascular morphogenesis (38). Furthermore, previous reports have shown that, STAT5 is essential for AKT-p70 S6K activity during lymphocyte proliferation in patients with leukemias and lymphomas (36). Similarly, the P38 pathway has been shown to activate the mTOR-p70 S6K pathway during oxidative stress in mouse embryonic fibroblast cells which culminates in upregulation of antioxidative enzymes that assist in reactive oxygen species removal and thereafter increase cell survival (39). Also shown previously, JNK phosphorylates p70 S6K to induce osteoblast proliferation and differentiation of MC3T3 cells, and can crosstalk in other physiological systems, for example, during hepatocyte proliferation (30–32). A study by Miwa et al, in 2012, showed that AKT-mTOR-p70 S6K, ERK, and NF-κB were involved together in proliferation of osteosarcoma cells and these pathways could be inhibited by caffeine thereby decreasing tumor burden (40). We observe in our results that most of the pathways activated by JAG1 in the pediatric HBO cells lead to the phosphorylation of p70 S6K, downstream. These findings implicating phospho-protein signaling pathways, especially NFkB and AKT (downstream of PTEN) are consistent with our transcriptional profiling (Figure 3) and collectively implicate p70 S6K as potentially an essential downstream contributor to JAG1-induced, pediatric HBO cell-mediated bone regeneration. Therefore, we proceeded to determine if p70 S6K is an essential downstream target of the JAG1-induced NOTCH non-canonical signaling in pediatric HBO cells (Fig. 4).

### Inhibition of phosphorylated p70 S6K leads to inhibition of JAG1-induced mineralization of pediatric HBO cells:

**Figure 5:**
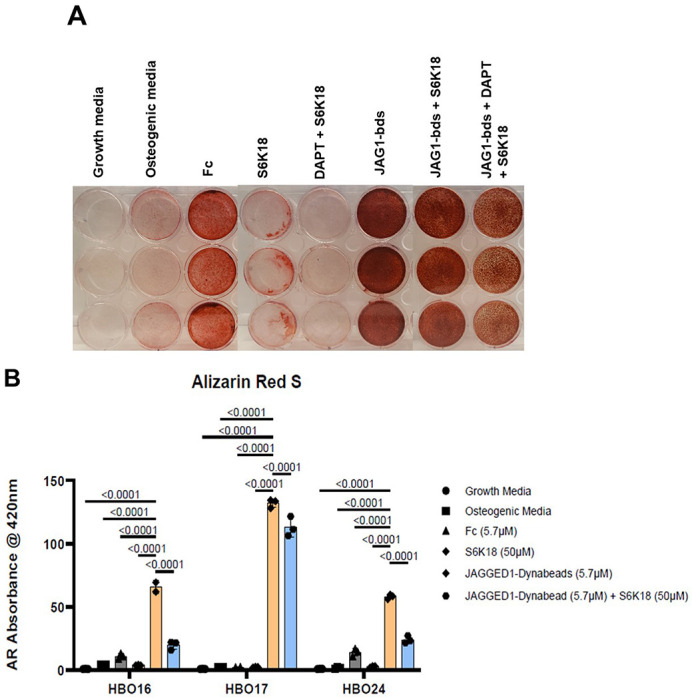
p70 S6K is an essential target during JAGGED1-induced mineralization of HBO cells: HBO cells were treated with growth media alone, osteogenic media alone or with Fc (5.7 μM), S6K18 (a p70 S6K phosphorylation inhibitor) (50 μM), DAPT (15 μM), JAG1 (5.7 μM) alone or in combination with S6K18 (50 μM) or S6K18 (50 μM) + DAPT (15 μM). The cells were half-fed every 5 days. On day 21 cells are fixed with 50% ethanol and thereafter, stained with 1% Alizarin Red S. (B) Alizarin Red S dye was extracted from stained cells using a 1:10 dilution of acetic acid and water, and the absorbance was read at 420nm. (5.7 μM). Data represents mean n = 3 ± SD per cell line with p-values indicated.

As shown earlier, we measured significantly increased ALP production as well as mineralization in JAG1 treated samples compared to all other treatments. We also showed an increase in the phosphorylation of p70 S6K, and according to multiple reports in literature, phosphorylation of p70 S6K occurs downstream of multiple pathways (AKT, JAK-STAT, and P38) that we identified in JAG1-induced pediatric HBO cells (36, 39, 40). Thus, we next tested whether the phosphorylation p70 S6K is an essential downstream target of JAG1-NOTCH during HBO cell mineralization. HBO cells were treated with growth media alone, osteogenic media alone, or Fc-Dynabeads (5.7 μM) as negative controls, and JAG1-Dynabeads (5.7 μM) with or without S6K18, an inhibitor of phosphorylated p70 S6K (41), in the presence of osteogenic media. On day 21, the cells were stained for mineralization using Alizarin Red S stain. As shown in Fig. 5, we observed that negative controls did not induce mineralization of the HBO cells while JAG1 induced significantly higher levels of mineralization, as expected. S6K18-treated samples showed partial inhibition of 50.2% of the JAG1-induced mineralization (p = 0.0015) and not 100%, possibly because the NOTCH canonical pathway was not inhibited in these samples. Inhibition of JAG1-induced mineralization by S6K18 treatment alone was significant compared to mineralization induced by JAG1 alone suggesting that the phosphorylation of p70 S6K is an essential event downstream of JAG1-NOTCH in JAG1-stimulated HBO cells.

## 4. Discussion:

We and others have previously demonstrated that JAG1 exhibits osteo-inductive properties in murine cell lines (1, 37, 42–44). JAG1 also induces the expression of pre-osteoblast genes like *Runx2* in murine CNC cells, even when the canonical NOTCH pathway is disabled using DAPT (1, 28), and further promotes CNC cell commitment to osteoblast differentiation via a JAG1-JAK2 non-canonical signaling pathway (1, 43). Thus, we hypothesized that JAG1 can induce osteoblast differentiation and mineralization of pediatric human bone osteoblast-like (HBO) cells, and the delivery of JAG1 with pediatric HBO cells constitutes an effective treatment for inducing bone regeneration in a pediatric craniofacial bone loss model.

To enhance the clinical translatability of this study, our lab derived human bone-derived osteoblast-like (HBO) cells by collagenase digestion of human pediatric fibulas bones. JAG1 has previously been shown to induce survival and proliferation of human mesenchymal stem cells, which are osteoblast precursors and various other human cell types, for example LNCaP which is a prostate cancer cell line, glioma cells, and intestinal epithelial cells during progression of colorectal cancer (45, 46). Osathanon et al, showed that tissue culture plate surface-immobilized JAG1 stimulated osteoblast proliferation and differentiation in iliac bone-derived cells (47). As seen in Figure 1A & 1B, we also observed increased mineralization of the JAG1-treated HBO cells compared to other controls (growth media alone, osteogenic media alone, and Fc-Dynabeads) in HBO cell lines obtained from 7 different pediatric fibular samples. This suggests that JAG1 reliably induces the human osteoblast-like primary cell expansion and differentiation in multiple human bone cell lines. These results suggest that the HBO cells behave similarly to the CNC cells *in vitro* and applying these humanized experiments to critically sized defects will test JAG1 as a potential bone regenerative therapeutic.

To confirm that JAG1 induces pediatric HBO cells to facilitate osteogenesis *in vivo*, as it did *in vitro*, we tested this strategy using an *in vivo* model of repair using murine craniofacial defects. As shown in **Supplemental Figure 3A & 3B**, PEG-4MAL hydrogels encapsulating JAG1-Dynabead and pediatric HBO cells were implanted in critically-sized parietal bone defects (4 mm) in athymic nude mice (NOD-SCID), which were used to prevent rejection of human cells. Four weeks later the mice received a second dose of treatments (**Suppl Fig. 3C**) via a transcutaneous injection to maintain the osteogenic signaling. Intermittent treatment with bone-regenerative therapeutics, like Parathyroid hormone (1–34), (48, 49) has been shown to lead to anabolic increase in bone mineral density. The ORTHOUNION clinical trial which aimed at enhancing bone healing in long bone nonunion fractures focused on testing the treatment involving two different sequential doses of expanded bone marrow-derived mesenchymal stem cells (50). Eight weeks after the initial dose, the regenerated bone volume was measured using MicroCT. As seen in Figure 2B, the fold change of bone volume regenerated by JAG1 in presence of DAPT (fold change:1.6, normalized to cells alone treatment) was comparable to JAG1 alone treatment (fold change: 1.5, p = 0.9583), and was significantly higher compared to the cells alone treatment group (p = 0.0038). The inhibition of NOTCH canonical signaling in mesenchymal stem cells using DAPT has been shown previously to enhance osteogenesis (51). A rheumatoid Arthritis C57BL/6 SCID mouse model carrying a human TNF transgene when treated with intermittent doses of DAPT showed improved bone regeneration (52). This supports our current results that JAG1 induces bone regeneration independently of NOTCH canonical signaling in the HBO cells. However, structural and mechanical properties of the bone will require characterization in the future using biomechanical testing.

As shown in our previous publications, JAG1 induced osteoblast commitment of murine CNC cells via a NOTCH non-canonical pathway and in our current data, it is observed that JAG1 induced HBO cells to regenerate bone and repair cranial defects even in the presence of a NOTCH canonical pathway inhibitor (DAPT), which suggested that the JAG1-induced pediatric HBO cell-facilitated bone regeneration occurs via a NOTCH non-canonical pathway. Therefore, to identify the NOTCH non-canonical pathway targets that are activated by JAG1 in HBO cells we isolated RNA from the JAG1-treated HBO cells, subjected it to RNA sequencing and performed pathway analyses on the data obtained. The data showed that a total of 448 genes were up-regulated, and 435 genes were down-regulated in JAG1 and JAG1+DAPT groups compared to no treatment and thus these genes are involved in the non-canonical NOTCH signaling pathway (Figure 3B, **Supplementary Table 1**). Some of the genes that are upregulated as part of the non-canonical NOTCH pathway are known to be involved in osteoblast commitment (RUNX2), matrix remodeling (MMP3), and diverse cytokines and chemokines (CCL5 and CXCL1). RUNX2 is commonly known to be expressed by osteoblasts as a sign of their recruitment into the lineage (53). MMP3 is also abundantly expressed by osteoblasts and is an important regulator of bone remodeling. MMP3 is an enzyme that is essential in the processing of collagen on bone surface, which in turn is necessary for osteoclast recruitment and bone resorption (54). Chemokine CCL5 is abundantly expressed by osteoblasts, and it is also involved in recruitment of osteoblast progenitor cells (55). PTH1/PTHR1 induces the differentiation of osteoblasts, and it has been shown that CXCL1 serves as an intermediate during this process (56). Gene ontology analysis of the up-regulated genes in the non-canonical pathway revealed significant enrichment of GO Terms associated with RUNX2, cytokine signaling, and cell cycle as described earlier are involved in osteoblastic cell proliferation, recruitment, and differentiation leading to bone remodeling. NF-κB was also upregulated and it is known to be a radiation-induced pro-survival factor in human osteoblastic cells (57). More interestingly, the PIP3 activating AKT signaling pathway was upregulated by JAG1 treatment, suggesting that JAG1 can activate NOTCH non-canonical signals via the AKT pathway. Collectively, these data reveal that JAG1 has a profound NOTCH non-canonical effect on HBO cells that stimulates HBO cell-osteoblast commitment and differentiation, and HBO-cell induced bone formation.

We also obtained lysates from HBO cells treated with JAG1 in the presence and absence of DAPT and subjected them to Luminex-based multiplex assays. The Luminex-based assays demonstrated that JAG1 induces the phosphorylation of various signaling targets, including STAT5, AKT, P38, JNK, NF-κB, and p70 S6K, between 15 to 30 minutes, as shown in Figure 4. As discussed earlier, prior studies emphasize the importance of non-canonical signaling, crosstalk between NOTCH, and other cellular signaling mechanisms (38) These studies show that STAT5 is essential for AKT-p70 S6K activity during lymphocyte proliferation in patients with leukemias and lymphomas (36), and that AKT-mTOR-p70 S6K, ERK, and NF-κB were involved together in proliferation of osteosarcoma cells (40). JNK was previously found to phosphorylate p70 S6K to induce osteoblast proliferation and differentiation of MC3T3 cells (58–60), and that the P38 pathway has been shown to activate the mTOR-p70 S6K pathway during oxidative stress in mouse embryonic fibroblasts (39). Taken together, our results and the results of others demonstrated that most of the pathways activated by JAG1 in the HBO cells converge at p70 S6K, making it a potential major contributor to bone regeneration caused by JAG1-induced HBO cells. Therefore, we proceeded to confirm that p70 S6K is an essential target of the JAG1-induced NOTCH non-canonical signaling in HBO cells (Fig. 5). p70 S6K is an enzyme that phosphorylates the S6 ribosomal protein to initiate protein synthesis which supports growth, proliferation, differentiation and glucose homeostasis of cells (61–63). We found that JAG1-induced mineralization in HBO cells was predominantly (50.2%) inhibited by S6K18, an inhibitor of the phosphorylation of p70 S6K, possibly because the NOTCH canonical pathway was still active in these samples. However, the predominant inhibition of JAG1-induced mineralization caused by S6K18 treatment alone was significant compared to that induced by JAG1 alone (p = 0.015), suggesting that the phosphorylation of p70 S6K is the predominant event downstream of JAG1-NOTCH in JAG1-stimulated HBO cells. Thus, p70 S6K is an essential event downstream of JAG1-NOTCH in JAG1-stimulated HBO cells.

Studying the mechanisms by which JAG1 induces osteoblast commitment and bone formation will enable new treatment avenues that involve the delivery of not only tethered JAG1 but also the JAG1-NOTCH non-canonical signaling intermediates themselves or their activators as powerful treatment options to induce bone regeneration in CF bone loss injuries. High-Throughput Screening for existent, FDA-approved drugs, compounds, and small molecules can be used to identify pharmacological activators of p70 S6K, as future directions of this study. These findings can provide powerful treatment options to induce bone regeneration in CF bone loss injuries and avoid the limitations with currently available therapies.

## Abbreviations:

CF: Craniofacial
BMP2: Bone Morphogenetic Protein-2
FDA: Food and Drug Administration
JAG1: JAGGED1
CNC: Cranial Neural Crest Cells
JAK2: Janus Kinase 2
STAT5: Signal Transducer and Activator of Transcription 5
HBO: Human Bone-derived Osteoblast-like (HBO) cells
p70 S6K: Ribosomal Protein Subunit p70 S6 Kinase
PTH (1-34): Parathyroid Hormone (1-34)
VEGF: Vascular Endothelial Growth Factor
FGF: Fibroblast Growth Factor
SDF-1: Stromal Cell-Derived Factor-1
TGFβ2: Transforming Growth Factor- Beta 2
PRP: Platelet-Rich Plasma
*Hes1*: Hairy and Enhancer of Split 1
Hey1: Hairy/enhancer-of-split related with YRPW motif protein 1
*Runx2*: Runt-related Transcription Factor 2
PBS: Phosphate-buffered Saline
(PEG)-4MAL: Maleimide end-functionalized four-arm Poly (ethylene glycol)
HEPES: 4-(2-hydroxyethyl)-1-piperazineethanesulfonic acid
NOD-SCID: Non-Obese Diabetic/Severe Combined Immunodeficiency
μCT: Micro Computed Tomography
BV: Bone Volume
DEXA: Dexamethasone
βGP: Beta – Glycerophosphate
DAPT: N-[N-(3,5-Difluorophenacetyl)-L-alanyl]-S-phenylglycine t-butyl ester
ANOVA: Analysis of Variance
SD: Standard Deviation
DMEM: Dulbecco’s Modified Eagle’s Medium
FBS: Fetal Bovine Serum
AKT: “Ak” in Akt refers to the AKR mouse strain that develops spontaneous thymic lymphomas. The “t” stands for ‘thymoma’, Protein Kinase B
P38: p38 mitogen-activated protein kinase
JNK: c-Jun N-terminal Kinase
NF-κB: Nuclear factor kappa B
WNT: Wingless-related Integration Site
mTOR: Mammalian Target of Rapamycin
MC3T3: Osteoblast precursor cell line derived from Mus musculus (mouse) calvariae
LNCaP: Cell line derived from a metastatic lymph node lesion of human prostate cancer
